# An Intelligent Low-Power Displaying System with Integrated Emergency Alerting Capability

**DOI:** 10.3390/s19030666

**Published:** 2019-02-06

**Authors:** Marius Vochin, Alexandru Vulpe, Laurentiu Boicescu, Serban Georgica Obreja, George Suciu

**Affiliations:** 1 Telecommunications Department, University POLITEHNICA of Bucharest, Bucharest 61071, Romania; alex.vulpe@radio.pub.ro (A.V.); laurentiu.boicescu@elcom.pub.ro (L.B.); serban@radio.pub.ro (S.G.O.); george@beia.ro (G.S.); 2 R&D Department, Beia Consult International, Bucharest 41386, Romania

**Keywords:** ePaper, iBeacon, alerting system, indoor positioning, low power display

## Abstract

Integrated communication infrastructure has become a must-have facility for modern public buildings and offices. To cover this need, several commercial products exist on the market, but most of them require advanced technical skill to operate, while others require manual and time-consuming operations. This work proposes an intelligent displaying and alerting system (called SICIAD), implemented over an integrated communication infrastructure with support for wireless ePaper and iBeacon technologies to enhance displaying static and dynamic information, as well as to ease the indoor orientation of guests. A centralized display management console is implemented, as well as procedures for automatically displaying different types of notifications. An Android mobile application is developed which enables indoor user location and guidance. The system targets educational and research institutions but could also cope with public institutions such as museums and hospitals. Remote authentication is supported in research facilities through eduroam technology, access being provided by the user’s distant institution of affiliation. Secure multiple-level access to the system is provided to users, from guests to system administrators, based on locally defined policies. Functional validation and performance evaluation aspects are presented for the proposed system.

## 1. Introduction

With the extensive usage of technology in all aspects of life, the need of an intelligent, integrated, sustainable, and easily managed system for digital and up-to-date room signage for offices, meeting rooms, and conferences has an increased importance for modern public and office buildings. The emergence of Internet of Things (IoT) and digital interactions using electronic paper (ePaper) [1] technology has marked a new phase of technological innovation in this direction. The electronic paper technology relies on ambient light reflection instead of using a backlight, as well as a screen that only consumes a significant amount of energy during the update phase. Such digital displays offer good visibility of information in all light conditions, with an increased image contrast and the benefit of a low power consumption. Important advantages are also brought by iBeacon technology, which relies on the Bluetooth Low Energy (BLE) standard to create stationary constellations of low-power beacons which can be used to determine the indoor position of mobile terminals or signaling points of interest [2].

The use of these technologies requires extensive computer programming skills to access and manage displayed information, since they are still relatively new. The current level of technology usually relies on the user to configure and manage different displays or write advanced scripts for mastering multiple information flows and dynamic update of these displays.

Display and notification solutions based on wireless ePaper are offered by multiple vendors. 

The solutions are, usually, based on the following elements:

- display, which can be either ePaper or liquid-crystal display (LCD);
- communication network, which can be based on Wi-Fi, 3G, or Bluetooth/ZigBee;
- content management and publishing software.

Several systems are focused more on displaying static content and less on dynamic content. There also exist on the market digital signage applications which focus on enhanced content, both static and dynamic, but they require a more complex infrastructure, so a low power consumption or flexible infrastructure is no longer the target, as will be shown in Section 2.

The integrated solution proposed in this work, the SICIAD research project, was started as a response to the identified technical needs of an important telecommunication company that could not be addressed by current commercial solutions on the market. The SICIAD system brings significant advantages to the end users by offering a unified developed web console as a management and control interface for ePaper displays, completed with indoor location services and iBeacon advertising. The Wi-Fi communication infrastructure on which SICIAD is built is implemented to offer customizable and reliable services, together with enterprise-grade encryption and secure remote user authentication.

This article is structured as follows: Section 2 presents some background on existing dynamic display and advertising solutions, Section 3 details implementation aspect of the SICIAD research project. Functional validation and performance results are given in Section 4, while relevant implementation and configuration details are discussed in Section 5. Section 6 presents the conclusions.

## 2. Dynamic Display and Advertising Solutions

The iBeacon communication standard [3] is a protocol implemented by Apple over Bluetooth Smart technology and can facilitate the development of location-based applications. A hardware device can send iBeacon radio signals to notify other mobile devices of its location. When a mobile device receives a beacon signal, it can use it to estimate the proximity to that beacon and the accuracy of the proximity estimation (ranging).

iBeacon devices use Bluetooth Low Energy (BLE) to broadcast unidirectional beacons in the 2.4 GHz frequency band. BLE is designed for low energy requirements and uses the wireless personal area network (WPAN) technology to transmit in its proximity. 

Electronic paper can be considered as a fixed or even portable display medium that can be remotely written on and refreshed multiple times in order to display new content, which can be retrieved from several sources or created with a device such as an electronic pencil (stylus). Therefore, ePaper can be defined as a display technique that simulates the appearance of text written on a traditional physical paper, offering a wider viewing angle than light-emitting displays and excellent readability in ambient light. Electronic visual displays can enhance appliances such as electronic shelf labels (ESLs), digital signage, time schedules for public transportation, billboards, portable signs, electronic newspapers, and even e-book readers. ESL solutions such as DIGI T@POP, [4], Altierre Anima, SES-imagotag, or Pricer bring a unique point of purchase pricing solution for retailers that allows operators to monitor and execute strategic pricing promotions conveniently. However, they require dedicated proprietary hardware, and the software provided can only be used for ESL displaying purposes. Additionally, pricing could not be identified for these solutions at the time of writing this document; one more disadvantage is that they offer limited extensibility and interoperability capabilities, due to their proprietary software restrictions. Digital room signage solutions are offered by vendors such as Visionect (with only 2 available sizes of 13″ and 32″, and a price of over one thousand euros per piece) and Visix. Cost effective standalone e-ink development kits are offered by vendors such as Pervasive Displays, Eink, or Adafruit; however, these solutions require relatively good programing skills and the use of individual display management, since they rely on opensource software and Arduino-based control hardware.

The LANCOM wireless ePaper solution is based on the principle that it only requires power when the displayed information is changed. A wide variety of functionalities and options for remotely displaying data are offered to the user in order to cope with their own customized use cases, such as radio-controlled signage at universities and real time content updating [5]. The wireless ePaper display eliminates the need for an external power supply or a physical network connection, as the devices are battery-powered and radio-controlled. Furthermore, the data transmission process can be protected by a 128-bit key, allowing secure encryption and authentication standards for eduroam [6]. The specifications of LANCOM ePaper displays indicate a battery life span between 5 and 7 years, if the displayed information is changed four times a day [7].

The most important step in operating the LANCOM ePaper solution is generating a template representing the image: communication of the template parameters, other XML configuration information, and wireless ePaper displays setup aspectsare discussed in [8]. This action can be automatically performed when the update is requested by a calendar management system (such as IBM Notes, Microsoft Exchange, or Google iCalendar, and the XML information is received by the wireless ePaper server. This server, which manages and monitors the access points and wireless ePaper displays, processes the received information code and arranges the display and the template, according to the requirement. The server then generates the data set used for delivering the image. The information from the XML is inserted after setting the layout to the template. Finally, the image that is delivered is sent to the wireless ePaper display.

Important benefits of the system proposed in this paper are information retrieving and displaying, and these are based on personal information management solutions such as Google Calendar or Microsoft Outlook in Office 365 [9]. An existing procedure is provided by and requires the use of an IronPython (http://ironpython.net/) script file, a special configuration file, and an XSL template. The three fundamental components can run separately, but they need to be installed in the same local network. The template and the image should be preferentially stored into an online directory. The script requires access to the mailboxes configured in Office 365. An account can be created from the configuration menu in Office 365, and an admin center meeting room can be selected. After setting up the meeting room preferences, another critical setting needs to be configured. The script and configuration file are a single logical body, since the script file contains the program code and the configuration file. The script file involves the code that queries the related information from Office 365, operates it, if required, and sends an update to the LANCOM wireless ePaper server.

However, the aforementioned procedure requires advanced technical knowledge from the user, who is supposed to create or adapt the IronPython script file, the special configuration file, and XSL template; therefore, the procedure is time-consuming and error prone.

**Figure 1:**
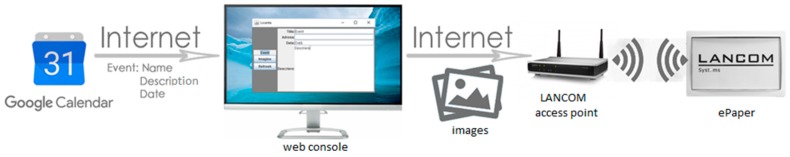
Typical SICIAD ePaper displaying scenario.

Our proposed integrated solution brings significant advantages to end users, by offering a unified custom developed web console as a management and control interface for online data sources, such as Google Calendar and ePaper displays, as can be seen in Figure 1.

## 3. SICIAD Implementation

The SICIAD project was proposed to capitalize on existing advanced technology available in a private telecommunications company’s premises, as described in [10].

Although the system is primarily designed for public institutions like universities or government buildings, some of its applications may include public transport, exhibition and commercial centers, museums, and both indoor and outdoor amusement parks, with the most relevant use cases presented in [11]. Any organization may benefit from an indoor positioning and orientation system, as well as a centrally managed displaying and alerting system.

### 3.1. SICIAD Management System Implementation

The first step in the design and development of the SICIAD system [12] has been to create the software modules necessary for controlling the available displays by interacting with the LANCOM API. Before more components of the system could be implemented, several experiments (discussed later in the paper, in Section 4.2) were performed to determine whether the low-power displays used can work within the system’s constraints (specifically as an emergency alerting system).

Since the ePaper server is written in Java, for consistency purposes, it was decided that SICIAD will also be based on the same programming language, making it a platform-independent software (depending only on the presence of a Java Virtual Machine to run and not on a specific operating system, it otherwise would have been compiled for). To simplify access to the software prototype, it has been implemented as a web application.

**Figure 2:**
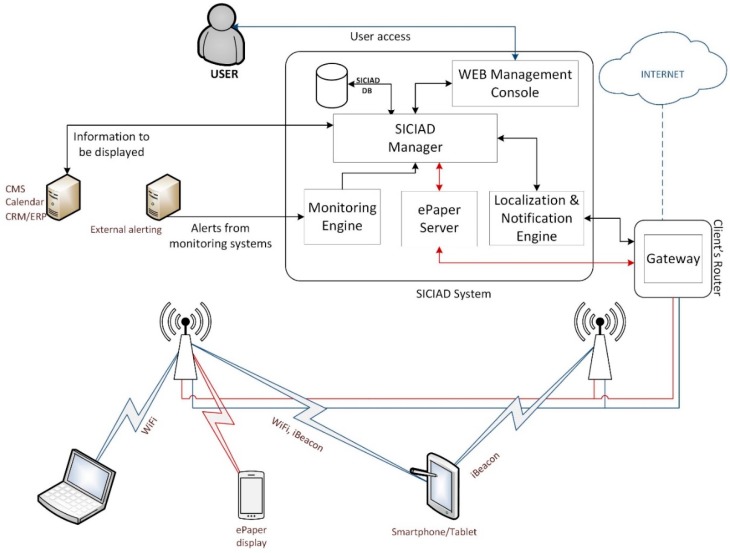
SICIAD conceptual architecture.

**Figure 3:**
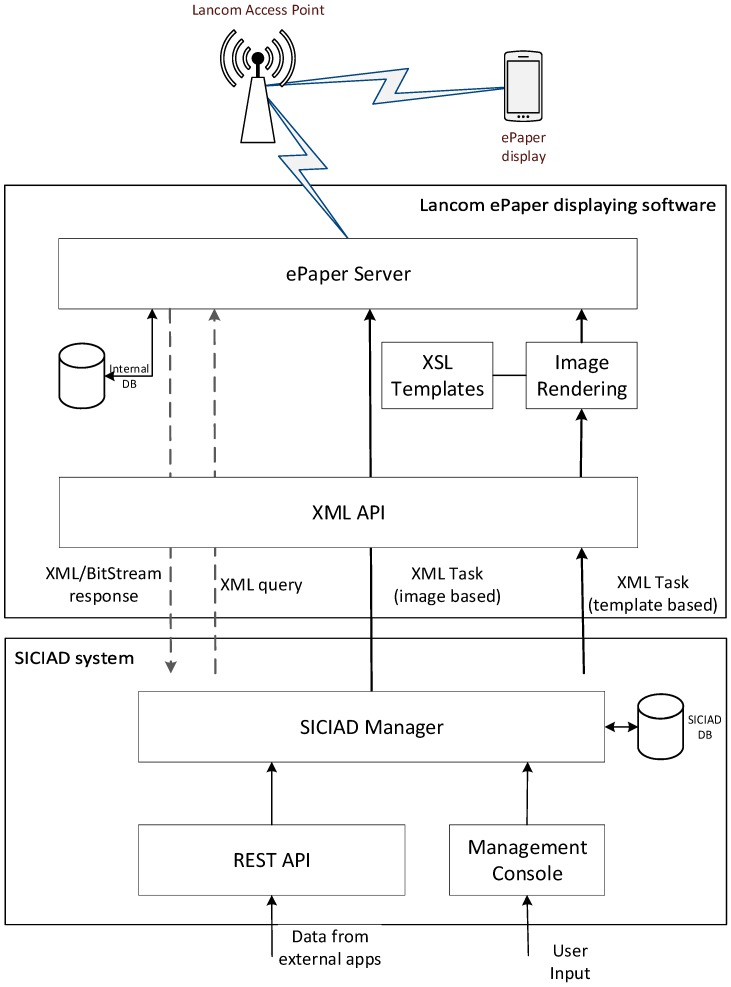
Informational flow of SICIAD-based ePaper display.

Figure 2 shows SICIAD’s conceptual architecture. All interactions with the wireless ePaper displays are represented with red lines in Figure 2, being performed through the ePaper server’s XML API, presented in Figure 3. A VPN (virtual private network—192.168.0.0/24) was deployed to overcome some of the drawbacks of the wireless ePaper displays, such as a lack of security in the communication channel between the server and the access points, as well as the requirement that both the server and the access points must be connected to the same local network. The architecture is further secured by restricting all access to the server and allowing users to interact exclusively with the SICIAD console.

**Figure 4:**
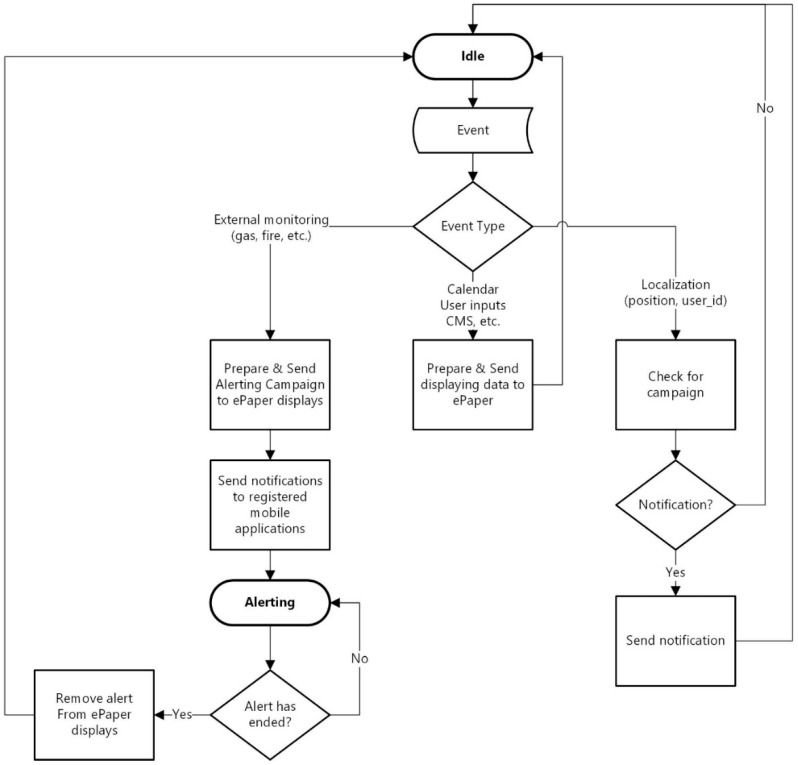
Logic diagram of the SICIAD Manager.

The system’s main components, presented in Figure 2 include the following:

- The SICIAD database, hosted on a MySQL server, used to store complete data regarding the ePaper displays (that includes performance data) and the information scheduled to be displayed on them.
- The Monitoring Engine, which is tasked with collecting data from external alerting systems or from the local sensors installed by the SICIAD system if necessary, and with sending alerts to the Management module, when the defined safety thresholds are exceeded.
- The Localization and Notification Engine, which provides the localization of the mobile terminal. It receives, from the application running on the mobile terminal, measurement data regarding the strength of the signals received from the BLE radio beacons and, based on it, computes the current position of the mobile terminal. The location information is sent to the Management module, which can use it to make decisions about notifications and alerts. The computed location information is also sent to the mobile terminal to use it for indoor positioning. The module is also responsible for sending the notifications and alerts generated by the management module to the mobile application.
- The SICIAD manager controls the system’s higher-level functions. It implements an HTTP client capable of making all ePaper server API calls and capturing (and identifying) response data, offering access to full HTTP information for analysis. An XML parser is also included, used to process all data received from the ePaper server. The parser is able to process performance data and convert it to CSV format, for further analysis with external tools. The module is responsible for sending image data to ePaper displays. These actions are triggered by calendar-type events, monitoring alerts, and location-based events. It is also responsible for generating notifications and alerts to the mobile application running on the mobile terminal, based on the received data from the monitoring engine and from the localization engine. The algorithm implemented by the SICIAD manager is represented in the logic diagram from Figure 4. The WEB Management console is designed as a web user interface for the system. It allows the user to configure wireless ePaper displays, external data sources, the monitoring engine, position-wise notification campaigns, and the schedule for display operations. It also gives the user access to reports on the status of the registered wireless ePaper displays and previously executed operations on them.

Figure 3 illustrates the flow of information related to ePaper displays, between SICIAD’s components, the Lancom ePaper displaying software, and external data sources through the dedicated REST API.

The REST API allows external applications to actively send data to the SICIAD system. This module was implemented in a later development phase, after several performance tests presented in the paper were performed. This is an add on feature of the system that has no impact on the performance data discussed in the paper.

Using the integrated HTTP client, the SICIAD manager communicates directly with the ePaper server (through its API), controlling the information loaded on the ePaper devices through two types of XML tasks: template-based (sending only text information to be rendered into images by the ePaper server, using a set of templates) or image-based (directly sending pre-rendered images). For the second case, images are encoded in Base64 format for XML compliance. Other XML queries allow the SICIAD manager to poll the ePaper server for information regarding the full status of all active ePaper displays and issued tasks, or even to download the displayed images.

Figure 4 presents the logic diagram of the main functionalities of the SICIAD Manager. The system configuration is done by the administrator via the Web Management Console. The ePaper infrastructure monitoring is also controlled via Web Console and is accomplished through the ePaper server. The main functionality of the Manager module is to provide data for displaying operation on ePaper devices. During normal operation, the SICIAD Manager instructs the ePaper server to display information on the ePaper displays, according to its configured schedule. The displayed information is obtained from the internal database, administrator inputs, or external applications such as a calendar (Google Calendar), content management systems (CMSs), enterprise resource planning (ERP) applications, or customer relation management (CRM) systems. The display operation is triggered by events that are going to happen in locations served by ePaper devices, events generated by calendar-type application, or by alerting events. For example, whenever the Manager receives a safety monitoring event (such as a gas or fire event), it interrupts normal operation and sends alerts to all registered mobile applications and ePaper displays, until the event ends. The second functionality of the SICIAD Manager is to generate notifications for the mobile application based on location-based events or monitoring alerts. Location-based events are generated by the Localization and Notification Engine, which permanently monitors the mobile device’s position, when the device is in a location of interest. If at that point of interest there is an event associated, the Manager will decide to send a notification to the mobile terminal.

All tests on the wireless ePaper architecture were performed through the SICIAD software prototype, and the results are discussed in the following chapters.

### 3.2. Mobile Indoor Location Application Implementation

Indoor positioning is a key function for systems offering location-based services. There are several approaches for indoor positioning [13,14]. Some of them are based on the processing of the received signal to determine the position of the receiving device. This family includes the triangulation-based methods, which use the received signal strength indicator (RSSI) values to determine the distance to the receivers and use triangulation algorithms to determine the position, and solutions involving complex antennas on transmitting and receiving stations, such as angle of arrival, time of flight, and phase of arrival [13,14]. However, determining the distance based on the RSSI values is difficult in an indoor environment due to multipath propagation. Additionally, complex antenna systems are expensive and are not appropriate for such applications. Other solutions use fingerprinting approaches, where a signal map of the indoor environment is built in advance and the position is determined by locating the current values of the received signals on this map. These solutions are relatively simple to implement, but they require an extensive measurement campaign and a careful calibration of the transmitted signal strength to build the signal map [13,14]. Additionally, they are prone to errors due to variations of the map determined by the environment changes. More recent approaches [15,16,17] are using unsupervised positioning schemes. They combine the fingerprinting approach with dead-reckoning based on smartphone sensors to estimate the position of users. To reduce the cumulative error, they establish landmarks using ambient signatures and users’ activity and use them to recalibrate the position. In [17], an unsupervised indoor localization scheme is proposed and evaluated successfully, with the obtained results showing a high localization accuracy. Such an approach has a more complex computational architecture and requires a training phase for precise localization of landmarks. On the other hand, its main advantage is that it has the ability to dynamically learn the environment. Additionally, they provide very accurate localization. 

However, for simplicity, a distributed solution based on a signal map approach was considered for the implementation of the indoor positioning of the mobile device. Among wireless technologies, BLE radio beacon technology fits indoor positioning solutions best because they provide a cheap and accurate positioning solution. Because SICIAD is built around WiFi and BLE technologies, the localization solution was designed to use those technologies, with the focus mainly on BLE. A signal map is built in advance by measuring the received signal strength at several points inside the location. The map is loaded on the central server. The mobile application measures the RSSI of the neighboring BLE radio beacons and sends the data to the server. The positioning application will apply the positioning algorithm with the signal map and the mobile measurement data as input. Once the position is determined, the location, and, if needed, notifications associated with the location, will be sent back to the terminal. At the same time, the system can update the ePaper displays nearby with the appropriate content.

A mobile application, developed in Android, enables the user to detect his/her location, based on the availability of Wi-Fi and iBeacon (Bluetooth Low Energy (BLE)-based radio packets) signal presence. The main functionalities of this software module are to capture BLE packets together with measured RSSI data, to extract location relevant information and send it to the SICIAD system for the localization process. Once the localization is performed, the mobile position and, if relevant, the notification or alerts related to current status are sent back to the mobile application.

Functional validation of the application is performed on commodity hardware such as LG K8 mobile phone with BLE 4.2 support and Android 6.0 operating system. The application tests if the device is Bluetooth enabled, whether the adapter is turned on, if multiple Bluetooth notifications are allowed, and whether access to the current location of the phone is allowed. As the instructions are parsed, a checkup is done to see if the permissions have been granted, and the user is asked to allow access. If all the conditions are met, the necessary processes are created, and the application continues to function. If something is missing, a corresponding error message will be displayed, and the application will stop.

**Figure 5:**
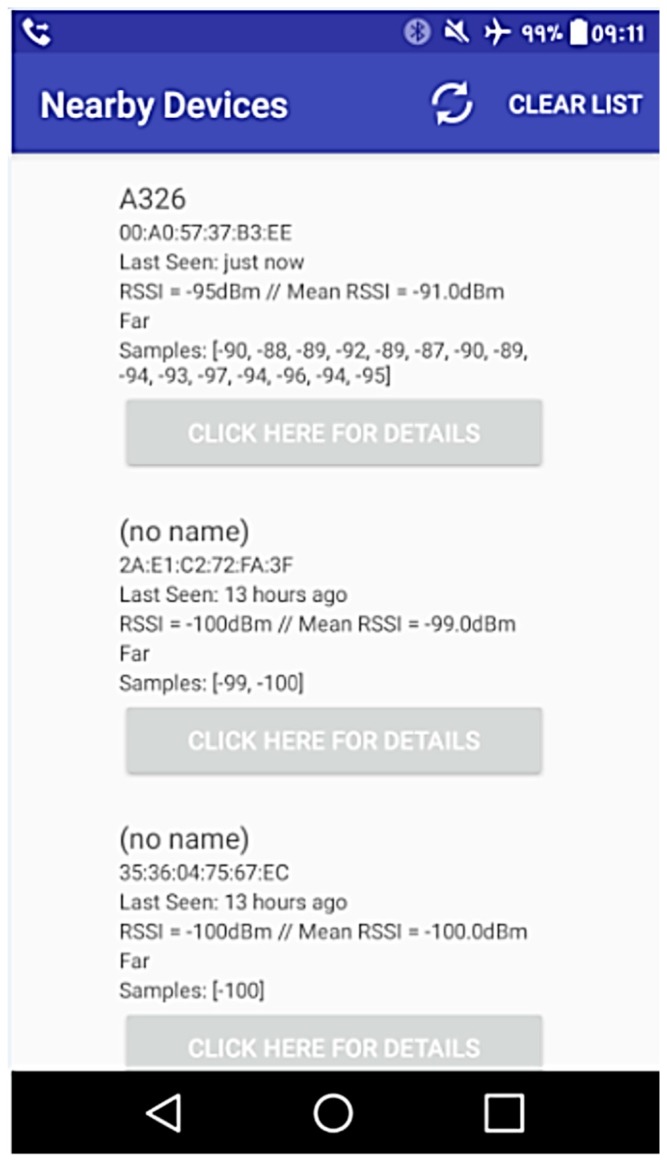
Mobile application beacon detection screen.

When scanning is started in debugging mode, a list of retrieved devices is presented with details (name, address, time since the last occurrence, current and average RSSI level, relative distance estimation, and RSSI mediated levels) and a details display button, as can be seen in Figure 5. If chosen by the user, details about the rooms in which LANCOM devices are located can be shown; moreover, other received BLEs beacons are displayed by the app, with less specific details. Unknown devices could be filtered before adding them to the list, resulting in faster application performance (no need to process their data).

## 4. SICIAD System Evaluation

**Figure 6:**
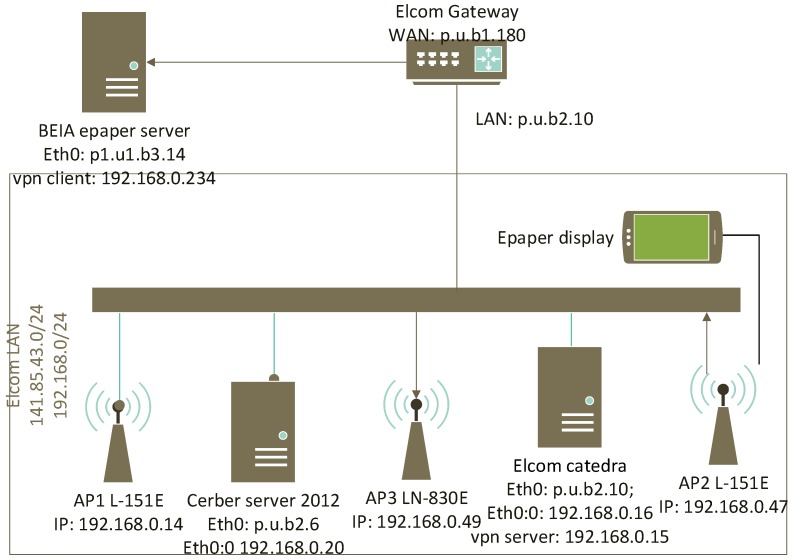
BEIA-UPB testbed.

The system was implemented over two premises located at BEIA and UPB (University Politehnica of Bucharest) which are interconnected as shown in Figure 6. The public IP addresses that are used for reachability between BEIA and UPB are marked as p.u.b.*.

The LANCOM ePaper server was installed on BEIA premises and a secure OpenVPN AES-256 encryption communication is ensured between the BEIA and UPB testbeds through the “Elcom” network Gateway (Cisco 2811 gigabit router), with an OpenVPN server running on a Linux Debian 9 machine named “Elcom Catedra”, located on UPB premises. The LANConfig software, which is a basic configuration tool provided by LANCOM, is installed on the Windows Server 2012 Cerber Virtual Machine used to configure the three access points and the ePaper displays.

The Wi-Fi communication services can be accessed unsecured with open authentication and no encryption or by using Wi-Fi protected access with enterprise grade AES encryption which is offered by the Elcom catedra FreeRADIUS server. The server can also work as an eduroam authentication server [18] in order to provide secure Wi-Fi access for the roaming guests if this is a required implementation feature.

The following sections investigate functional and performance aspects of the control and displaying systems, and, later, similar aspects are addressed regarding the mobile Android application that provides indoor location capabilities.

### 4.1. Power Management

In order to determine the battery behavior of iBeacons in the SICIAD architecture, we used a topology that resembles as closely as possible the network in the BEIA company building that would be used to implement iBeacons in the SICIAD architecture. The battery behavior is important, because it serves as a metric for determining the advertising interval of iBeacon. A small interval such as 100 ms (as recommended by Apple) would likely lead to a fast discharge of the beacon battery. A high interval (larger than 1000 ms) might lead to a drop in the positioning accuracy for applications such as indoor positioning. It is therefore important to establish the optimum advertising interval of the iBeacon packet for controlling the power consumption and maintaining the low-power assumption. 

We used battery models available in the EXata Network Simulator [19] to enable the analysis of discharge behavior under different system loads without time-consuming and expensive prototyping. The EXata Network Simulator was used in order to send iBeacon frames to receiving devices, and two battery models were used: the *Service Life* battery model [20] and the *Residual Life* battery model. A more thorough analysis of the topology used, simulation parameters and results can be found in [10].

**Figure 7:**
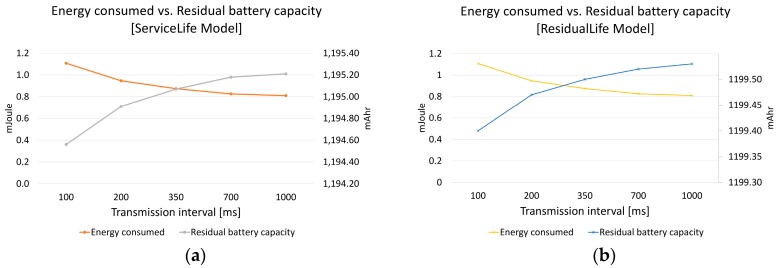
Energy consumed vs. residual battery capacity in (**a**) the ServiceLife model and (**b**) the ResidualLife model.

Figure 7 presents in a single chart the energy consumed and the residual battery capacity, using the ServiceLife model (Figure 7a) and ResidualLife model (Figure 7b). We notice that the consumed energy does not decrease linearly, and the rate of decrease is higher at lower transmission intervals (which was naturally to be expected). On the other hand, the residual battery capacity tends to increase as the transmission interval decreases. This means that the battery of the beacon will last longer when having a higher transmission interval.

Figure 7 illustrates that, by using the recommended transmission interval (100 ms), the battery would deplete in a very short time, but increasing the transmission interval to 1000 ms does not lead to a 10-fold drop in the discharge rate. The battery life lasts between 13 and 30% longer (depending on the battery model that was used).

Looking at the residual battery capacity together with the consumed energy curve, the recommendation is to choose an advertising interval of 300–350 ms, as this can accommodate both a good indoor positioning accuracy and a good battery discharge rate.

Battery life depends on several factors, such as operating environment, advertising interval, the presence or absence of sleep cycles, and many more. It is difficult to state precisely the amount of time necessary to deplete the battery, especially if we take into account that this is a simulation which uses two different models. However, if we consider an ideal operating temperature of 23 °C (73.4° F) and no sleep cycles, a 30% increase in battery life translates into an increase from 9.25 h to 12 h for battery life using the ServiceLife model, and an increase from 83 h to 108 h using the ResidualLife model, taking into account a battery of 1200 mAh and that the simulation time was 150 s. One must note though that this assumes continuous transmission; therefore, in a real life scenario, these figures would be closer to the current advertised battery life for beacons (estimated at over 3 years).

### 4.2. Displaying System Evaluation

Several experiments were performed in order to determine the delays that occur when updating the information on the ePaper displays from the wireless ePaper server. To that end, a testbed containing one LN-830E [21] and two L-151E [22] access points was used, together with WDG-1 (black and white) ePaper displays of 7.4″, 4.2″, and 2.7″ screen sizes. The access points were registered to a remote ePaper server installed on an openSUSE Linux machine located at the BEIA premises. The latest version of the LANconfig configuration software was installed on a virtual Windows Server 2012 machine, in order to be able to configure the access points, register, and update ePaper displays with the use of ePaper server software. 

**Figure 8:**
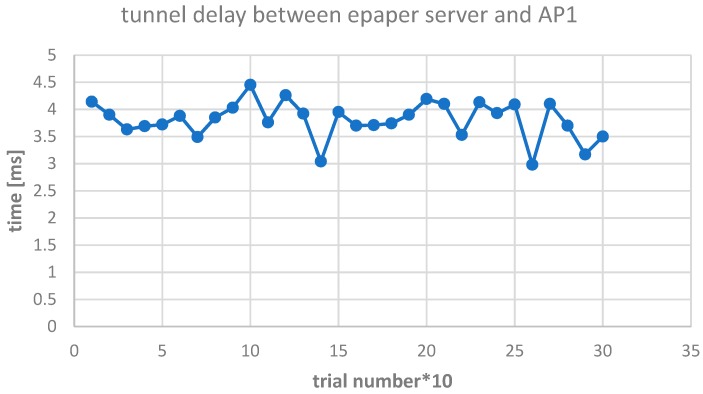
VPN (virtual private network) tunnel delay.

The Elcom testbed LAN was connected with the remote BEIA ePaper server through a VPN tunnel, as can be seen in Figure 6, since the ePaper server, access points, and ePaper displays are required to be connected in the same LAN, for licensing purposes. The delay introduced by the tunnel between the ePaper server and AP1 is represented in Figure 8.

We see that the delay has little variation, making the VPN between BEIA and UPB stable and without a significant influence on the next presented results.

Further testing was conducted in more strict conditions, at BEIA premises:

- seven ePaper displays were used this time, varying in size from 2.7″ to 7.4″ (including WDG-2 type, which can display information in black, white, and red);
- the wireless ePaper displays were placed in close proximity to the LANCOM access point, checking that there is no interference with Wi-Fi networks or other devices working at 2.4 GHz. In these conditions, the only possible radio interference would be the one between the displays.

#### 4.2.1. Displaying Delay Evaluation for Separate Image Loading

Depending on their size, LANCOM’s ePaper displays can store and switch between up to 8 or 12 images (referred to as pages by the ePaper server API). The approach offers a good degree of flexibility, allowing the system to switch between images already stored in the display’s memory and avoid congesting the low-power wireless channel with unnecessary data.

The images displayed on the ePaper devices can be controlled through three types of actions:

- switch page, instructing the device to display an image (page) stored in its memory;
- preload image, instructing the devices only to store images in their memory without directly displaying them;
- display image, directly displaying new images on the devices.

The performance indicators described in the following tests are directly measured by the ePaper server and illustrate the following:

- The image transmission time—the time it takes to physically transmit the image to the ePaper display, ignoring all other delays.
- The task duration, or the complete time it takes to successfully complete the task on the target device. For example, for display image tasks, the task duration includes the complete time to transmit the image to the wireless ePaper display (i.e., the transmission time), display the image on the device, and confirm the successful operation to the server. The task duration will also include any delays resulting from radio interference (between adjacent displays).

The test images were optimized for the target ePaper displays: compressed B/W PNG images were encoded in the Base64 format and sent to the displays using the LANCOM proprietary XML API, using the console mentioned in Section 3. The same system was used to download and interpret all performance information from the ePaper server.

The first performance test (Test 1) aimed to determine whether there are any differences between the relative performances of displays with different sizes. Same-size and similar-content images (300 bytes) were sent to all displays through *display image* tasks. The test results show a consistent 236 ms transmission time in all cases, regardless of the displays’ sizes. In all cases, in the absence of interference and transmission errors, the task duration is less than 15 s.

The second test (Test 2) performed at BEIA partner premises was envisioned as a stress test: six large images were tasked to be uploaded simultaneously to each of the seven ePaper displays (all connected to the same access point), using both preload and display directly tasks. Once started, the entire test took nearly 10 min to complete, with only 4 of the 42 tasks having failed. Images varied in size from 2.5 to 17 kB, proportional to the display sizes.

The results from Test 2 are included in Table 1 and confirm that the transmission times are proportional to image size.

#### 4.2.2. Displaying Delay Evaluation for Bulk Image Loading

The final, third test (Test 3) was designed in order to determine the relationship between the uploaded image size and the effective transmission size. To that end, optimized B/W images were generated, ranging from 1 to 25 kB, and were all uploaded to the same 7.4″ display. Each image was uploaded three times, but not before the previous task was finished.

It is important to note that results from Table 2 confirm that there is no difference in transmission times between preload and display tasks. The differences that can be noted, however, in the full execution time of the tasks relate to the display technology used. ePaper displays are slow, with refresh times in the order of seconds or even tens of seconds (the tests we performed indicate a 4 s refresh time for the 7.4″ display).

Tests 2 and 3 show the following:

- Each ePaper display polls the ePaper server for updates approximately every 15 s.
- The ePaper server recompresses all images received through the API. In most cases, the images recompressed by the server were slightly larger than the ones specifically optimized for this test, but there was one case where the server reduces the image by almost 20%.
- Transmission time increases in an exponential fashion, but for images with a size lower than 5kB the increase can be approximated as near-linear.
- Transmission time seems to remain constant for larger image sizes. For example, for image sizes between 10 and 30 KB, transmission time seems to be limited at 6.5 s. These performance metrics are provided by the ePaper server, and, since it is a proprietary solution, the above behavior cannot be further investigated.
- Although there are no other external interferences, ePaper displays still seem to interfere with each other, specifically during image upload operations. However, the interference is contained in the operational range of each access point.

Test 3 indicates that both Preload and Display tasks result in the same amount of information sent over the (proprietary) low-power wireless channel, to the ePaper devices. As shown in Table 2, the same transmission time is obtained in both cases. The only difference between the two types of tasks is that the latter involves the additional delays of changing the ePaper displayed image and confirming task completion.

**Figure 9:**
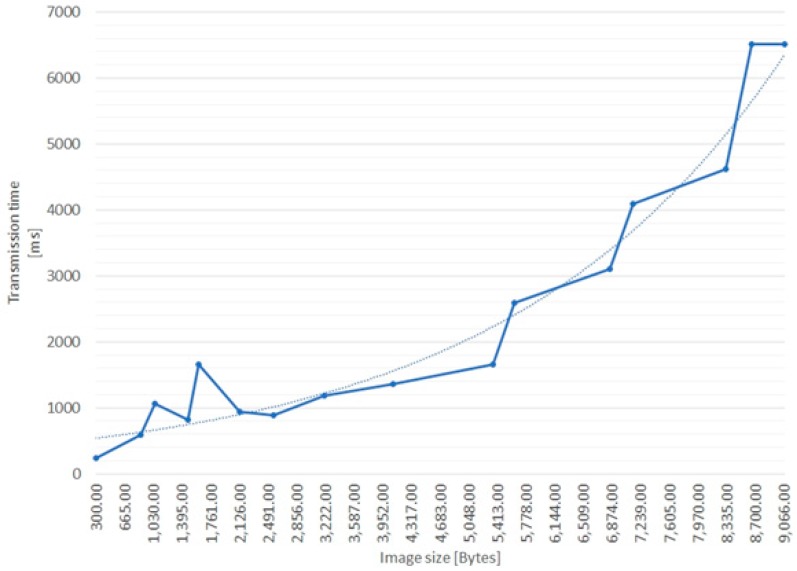
Image transmission time vs. size.

Figure 9 shows the dependency between the image transmission time (on the wireless channel) and the image size, suggesting that small images (less than 5 kB), optimized for B/W ePaper devices, could be transmitted in less than 2 s. In the absence of radio interference, such images could be displayed in less than 30 s.

Investigations were done in order to determine

- the interference between ePaper displays connected to the same AP,
- the impact of simultaneously displaying information on multiple displays, and
- the maximum number of displays that can be used for emergency alerts, with an acceptable delay.

An important performance aspect on the wireless ePaper display side configuration is the balance between transmission power and transmission time, together with the correlation between image size and transmission time/total duration. At the same time, it is possible to reduce transmission even further, through the use of the ePaper image rendering system.

### 4.3. Mobile Positioning Solution Evaluation

In this test scenario, the iBeacon capability was investigated with respect to indoor advertising, guidance and location. Commodity hardware was used at the BLE receiver part, meaning two types of smartphones (SM-G361F and, G930FD) and one laptop (HP Pavilion ×360 -13-u103nq) laptop. Different measurements were conducted on the testbed in the UPB campus, which is a reinforced concrete building, with 30-cm-thick walls. 

**Figure 10:**
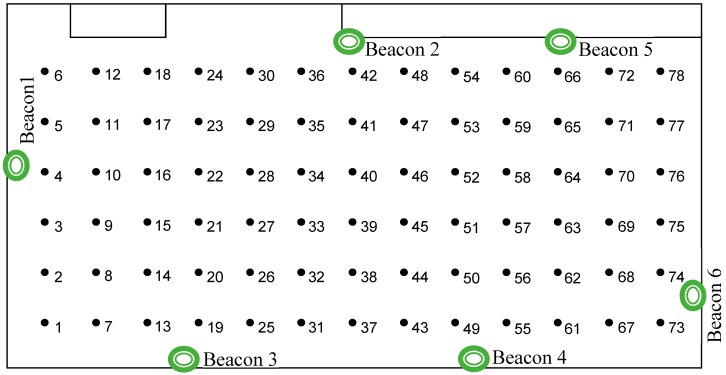
The experimental setup for localization solution evaluation.

To test the performances of indoor localization based on iBeacon technology, an experimental setup was developed: an empty laboratory room was used for localization experiments (Figure 10). The room has a surface of about 100 m^2^ (14 × 7 m). An empty room was considered in order to be able to study the localization performances in a near-ideal environment. In real life, the indoor environment would be more crowded with obstacles, increasing the variations of the received signal strength caused by multipath propagations. To build the signal map, a matrix of measurement points was deployed in the room. The measurement points were placed at a distance of 1 m from each other along the main directions of the room. As can be seen from Figure 10, there are 13 columns of six measurement points in the matrix. Six radio beacons from Kontakt.io were placed at about a 2 m height from the floor in the positions indicated in Figure 10. The transmit power for each beacon was set to −8 dBm.

To build the signal map, around 130 values of the RSSI parameter, measured by the mobile phone, were collected at each point of the measurement matrix, for each beacon device. The mobile phone was placed at a height of around 1 m from the floor. At each point, it had the same orientation. The RSSI measurement values were uploaded on the SICIAD server, where the localization solution is running. The localization solution is based on the K-nearest neighbors algorithm [23]. It receives the measured RSSI values in the current position from the mobile terminal. The algorithm computes the distance between the current RSSI values, at the point where the mobile device is located, and the RSSI values from the signal map. The first K nearest neighbors in terms of RSSI distance are determined. From the set of K neighbors, the one that is found more frequently in the set is chosen as the position point. A value of 15 was chosen for K. The Euclidean metric was used by the algorithm to compute the distance between the signal maps points and the current measurement point.

**Figure 11:**
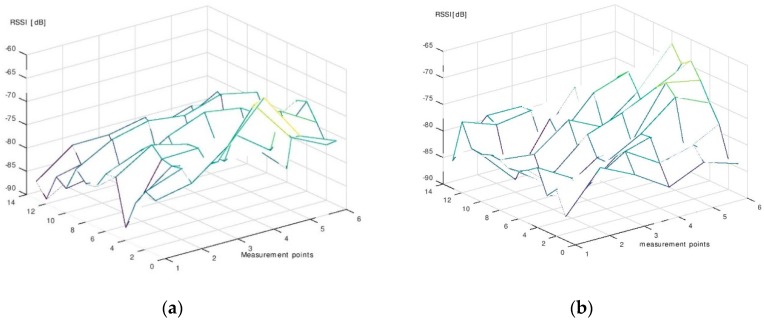
(**a**) The average RSSI—Beacon 1. (**b**) The average RSSI—Beacon 2.

**Figure 12:**
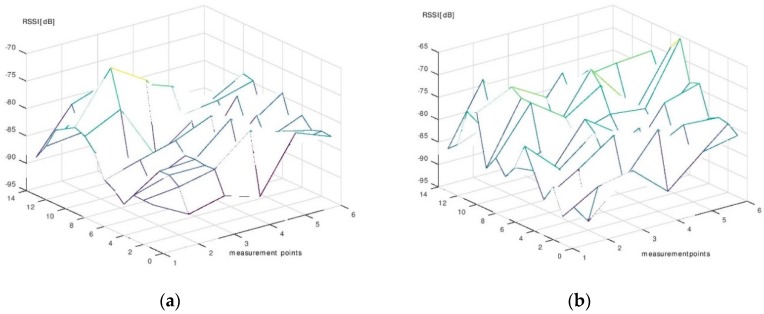
(**a**) The average RSSI—Beacon 3. (**b**) The average RSSI—Beacon 4.

**Figure 13:**
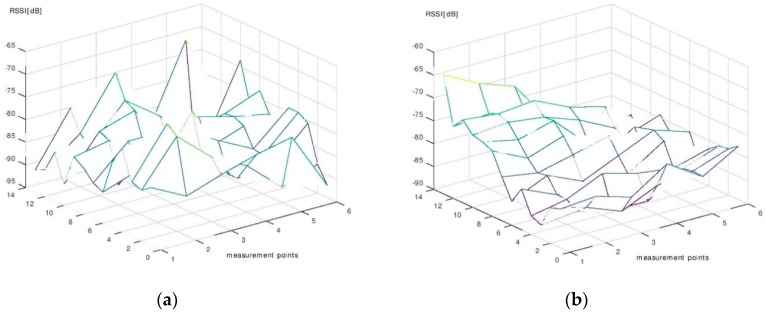
(**a**) The average RSSI—Beacon 5. (**b**) The average RSSI—Beacon 6.

The results of the measurement campaign performed during the phase of signal map construction are presented in Figure 11, Figure 12 and Figure 13. Each point on the graphs was obtained by averaging the 130 RSSI values obtained during the measurements for the signal map.

One can see that even the average signal presents rather random variations in some regions of the room. However, it can be noticed that, near the beacons’ locations, the signal’s average values are significantly higher than the ones obtained in farther regions. Based on this remark, we made the decision to use six beacons for localization. With six beacons, most of the room surface is covered with regions where the signal from one beacon is significantly higher than the signals from all other beacons.

In an initial phase of evaluation, only three beacons were used for the localization solution [24]. The results presented in [24] were obtained in the same location, with three radius network beacons configured with a −3 dB transmission power. Very poor localization precision was obtained with that setup. The mean localization error was around 5 m, which is very high considering the room dimensions. The reasons for such poor results are higher fluctuations in the power of the received signal, probably caused by the stronger reflected waves, the radio transceiver features of the radius beacons, the low number of radio beacons, and the low number of measurement values used to build the signal map. In the current experiment, the number of beacons and measurement values collected at each point was increased to improve the localization precision. The transmitted power was also reduced in an attempt to limit the effects of the reflected waves. Another modification was the replacement of the radius beacon devices with more performant ones from Kontakt.io. They have a better radio module, thus providing a more stable signal.

**Figure 14:**
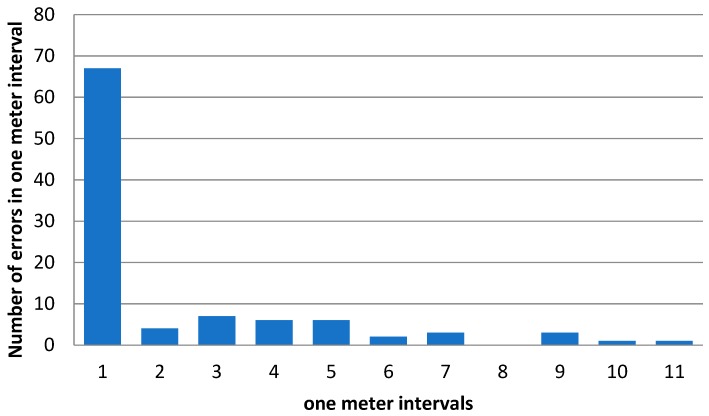
Distribution of localization error.

After the signal map was built for the new setup with six Kontakt.io beacons, the solution was evaluated by computing the localization error in randomly selected locations inside the room. The locations were randomly selected from the pool of measurement points used to build the signal map. To obtain relevant statistics, 100 tests for the localization error were performed. Since the locations for evaluation were selected from the pool of measurement points used to build the signal map, the localization error can be zero. The algorithm can precisely determine from the matrix the point where the mobile device is placed during tests. If the mobile is placed in any other location of the room, a zero value for the localization error will be less likely, resulting in a higher average localization error. With this setup, the average localization error, for the one hundred evaluation points, was around 1.3 m. This result represents a major improvement compared to the three-beacon version of the solution. The distribution of the localization error is presented in Figure 14.

The graph represents the number of times the localization error was contained in one of the 1 m intervals: [0-1), [1,2), and so on. The distribution shows that the localization works well most of the time, high localization errors being sparse events.

Based on the available signal maps, built for the six Kontakt.io beacons with a transmission power of −8 dB, the localization error was evaluated in the case of fewer beacons. The results, when only five beacons are considered for the localization process, are presented in Table 3. The values in the second column of the table represent the average localization error when five beacons are used to build the signal map. The first column specifies the beacon missing from the six radio beacons used for localization.

The results in Table 3 show that the average localization error is approximately the same as that obtained in the case of six beacons. For Beacon 6 the localization error is slightly better. This could be justified by the fact that Beacon 6 is very close to Beacons 5 and 4, which can cause some overlapping of the regions with a high RSSI from only one beacon.

The number of localization beacons was modified, and the results are presented in Table 4. As was expected, the localization error increased while the number of beacons decreased. Additionally, one can notice that four beacons is the minimal number for providing acceptable localization accuracy considering the size of the room used in the experimental setup. By using five beacons with appropriate positions, it is possible to achieve precise localization. With three beacons, the average localization error is still good, better than the one obtained in the initial investigation phase with the same number of beacons and the same configuration, but with a higher transmission power of −3 dB. This proves that the transmission power must be also considered when an indoor localization solution is designed. 

The results show that, by using an appropriate number of beacons and optimal positions, a relatively precise indoor localization can be obtained with iBeacon technology. However, these results were obtained in optimal conditions. More realistic conditions have an influence, such as the presence of people in the room, other objects that could determine attenuation, multipath propagation, and other unwanted effects on the beacons’ signals. Signal map measurements should be repeated in order to deal with new significantly sized static objects introduced in the room. The major drawback of this solution is that it requires the construction of the signal map during the initial setup of the system, which can be a time-consuming process. Additionally, the modifications in the signal map due to the variable environment are not addressed by the present solution. To get rid of these shortcomings and to further improve the localization precision, an unsupervised indoor localization approach, like the one in [17], should be considered.

## 5. Discussion

SICIAD focuses mostly on centrally managing the information displayed on the ePaper devices, leaving all management functions to the already implemented LANCOM tools. The system uses iBeacon devices integrated in the LANCOM access points to provide indoor location services. However, the use of standalone BLE beacon emitting devices such as the ones provided by Kontakt.io is also considered, to enable a more accurate location of users or provide a more refined point of interest advertising.

For licensing purposes and optimizing operational costs, the LANCOM ePaper server was installed at the BEIA partner premises, access to it being tunneled, but the impact of the delay induced by the tunnel over the response time of the system can be considered negligible. However, a production installation of the system might bypass any tunneling between the ePaper server and the access points, by collocating all the components of the system in the same LAN, in order to exclude extra delay and other potential issues introduced by the tunneling technique. In such a case, the availability and performance would increase. There could, however, be situations when a potential customer might want to deploy the system in a physically separated location from the ePaper server.

Consistent efforts were made for system implementation and functional validation scenario testing, but security optimizations and performance improvements were also considered.

In order to maintain the real-life relevance of the obtained data, commodity hardware was used in test scenarios. A professional mobile BLE receiver was used in order to improve indoor location awareness precision, but this questions the relevance of the data obtained in the context of commercial and industrial applicability.

While the iBeacon transmitters integrated at the wireless access points used can enable location-based services, accurately determining each users’ location may require additional, battery-powered BLE beacons. Such a network (or constellation) of beacons could provide a more performant indoor guidance system, due to its effectiveness at a range of several meters (compared to several centimeters for NFC tags). Moreover, although the investment in beacon devices may be significant, the already widespread use of compatible smart devices may reduce the necessity of other hand-held devices.

Based on the experiments described in Section 4.2, it can be said that an average 15 s delay may be deemed acceptable for displaying alerts on single devices. However, when working with significant numbers of wireless displays, the response time may rise to unacceptable levels (several minutes if only the average case is considered, tens of minutes in the worst-case scenario—one must consider concurrency issues on the wireless channel).

In order to respect the constraints of displaying emergency alerts, the messages can be preloaded on the displays, and the system can then use a more rapid switch operation (which only takes a fixed 59 ms, a short enough time to avoid errors due to radio interference). Even then, the simultaneous update of a large number of devices may rise to undesired levels, in the case of an emergency.

Table 5 shows SICIAD’s optimal operating parameters, based on the test results discussed in Section 4.

The Lacom ePaper server has API functions for both directly displaying information (display image task, discussed in Section 4) on the ePaper displays, and preloading in it advance and displaying it per user request (preload image and switch page tasks). However, tests show that the latter should be used if the information is available in advance. In order to obtain the targeted 30 s response time for the ePaper displays, it is recommended that images are displayed with a maximum size of 5 KB. The recommended method for loading images on ePaper devices is the preload image task regardless of image size, but images larger than 5 KB should never be directly displayed.

## 6. Conclusions

The system presented in this work implements an integrated communication infrastructure that offers dynamic display capabilities using ePaper technology and that enables indoor location-based services such as visitor guidance and alerting using iBeacon-compatible mobile devices. SICIAD builds on the LANCOM ePaper solution, simplifying the management and automation of all display operations. All performance tests on the ePaper infrastructure are performed through the implemented SICIAD system.

Being based on the BLE standard, iBeacon technology can potentially operate with almost all commercial off-the-shelf smart mobile terminals, providing a cost-effective solution for an indoor positioning system. In combination with a smartphone application and a wireless communication system, BLE can enable the advertising and distribution of location-based content.

Performance measurements show that the ePaper display’s response times are relatively short, being suitable for the proposed use cases. Enhancements are needed to guarantee rapid response times in case of emergencies. In this regard, it has been established that preloading specific messages to the display, and only switching to the corresponding pages is then desired over the much slower image upload.

The batteries offered by the manufacturer of the ePaper displays are sufficient for most of the use cases, and it is easy to replace them for indoor applications. For outdoor applications, ePaper systems can be backed up via solar photovoltaics panels and, due to their low power consumption, may function entire seasons without sunlight, offering a long-term solution for displaying information in hard-to-reach areas.

## Figures and Tables

**Table 1:** Transmission time, different display, and image sizes for Test 2.

Display Size	Transmission Time (ms)	Image Size (kB)
2.7″	885	2.5
4.4″	2124	6.3
7.4″	5015	17

**Table 2:** Display and preload operation delays.

Operation	Transmission Time (ms)	Task Duration (s)
Preload	5.015	73
Display	5.015	108
Switch page	0.059	21

**Table 3:** Average localization error in the case of five beacons.

Missing Beacon	Average Localization Error (m)
Beacon 1	1.533
Beacon 2	1.273
Beacon 3	1.622
Beacon 4	1.404
Beacon 5	1.417
Beacon 6	0.978

**Table 4:** Average localization error when the number of beacons is changed.

Number of Beacons	Average Localization Error (m)
6	1.305
5	1.533
4	1.983
3	2,437
2	3,630

**Table 5:** Optimal operating parameters for SICIAD.

Parameter	Value
Maximum ePaper image size	5 KB
Maximum page switching time	30 s
Optimal image loading task	Preload
